# Dissemination and Implementation of a Text Messaging Campaign to Improve Health Disparities among Im/Migrant Workers

**DOI:** 10.3390/ijerph20075311

**Published:** 2023-03-29

**Authors:** Ellie Cherryhomes, Shannon Guillot-Wright

**Affiliations:** 1 Department of Pharmacology and Toxicology, University of Texas Medical Branch, Galveston, TX 77550, USA; 2 Center for Violence Prevention, Department of Family Medicine, University of Texas Medical Branch, Galveston, TX 77550, USA

**Keywords:** text message campaign, health equity, commercial fishing, migrant workers, occupational health

## Abstract

The use of short message service (SMS) text messaging technology has grown in popularity over the last twenty years, but there is limited data on the design and feasibility of campaigns to reduce work-related injury, particularly among rural workers, non-native English speakers, and illiterate or low-literacy populations. Although there is a critical need for tech equity or ‘TechQuity’ interventions that reduce injury and enhance the wellbeing of under-reached communities, the barriers and benefits to implementation must be empirically and systematically examined. Thus, our team used D&I science to design and implement an 18-week texting campaign for under-reached workers with a higher-than-average risk of fatal and non-fatal injury. The experimental project was conducted with English-, Spanish-, and Vietnamese-speaking commercial fishermen in the Gulf of Mexico to test the design and feasibility, and messaging focused on preventing injury from slips, trips, and falls, as well as hurricane preparedness. The ubiquity of mobile devices and the previous success of texting campaigns made this a promising approach for enhancing health and preventing injury among an under-reached population. However, the perceived benefits were not without their barriers. The lessons learned included the difficulty of navigating federal regulations regarding limits for special characters, enrolling migratory participants, and navigating areas with limited cellular service or populations with limited accessibility to technology. We conclude with short- and long-term suggestions for future technology interventions for under-reached worker populations, including ethical and policy regulations.

## 1. Introduction

Commercial fishing is a diverse industry with an estimated 700,000 workers and 51,000 active fleets managed by eight regional fishery management councils in the United States (US) [1,2]. On average, commercial fishermen have a fatality rate 29 times higher than the average worker [3]. The Gulf of Mexico (GoM) is the second-most productive commercial fishing industry in the US and one of the most dangerous sectors for employment [4,5]. Moreover, in the US, im/migrant and seasonal workers support the commercial fishing industry and were considered essential workers during the COVID-19 pandemic. Rife with fatal and non-fatal injuries, GoM commercial fishermen face occupational and structural factors that impact their overall health and wellbeing. The hours of work for commercial fishermen are dynamic, with fleets working offshore upwards of weeks and some fishermen working with less than fours hours of a sleep during a 24-h period [6]. The risks include physical demands such as moving thousand-pound nets, operating with and around heavy machinery, and performing repetitive motions [7,8,9]. These risks are compounded by environmental factors such as dangerous waves, extreme heat, extended ultraviolet exposure, increasingly severe weather and natural disasters associated with climate change, and exposure to manmade disasters such as oil spills [10,11]. Structural factors that underpin workers’ social conditions and physical and mental wellbeing include high-occupational health risks, dangerous working conditions, low wages, and precarious employment.

Given these risk factors, occupational health trainings that take economic, cultural, and social factors into account need to be more widely adopted by occupational health researchers and practitioners or what the Centers for Disease Control and Prevention (CDC) and National Institute for Occupational Safety and Health (NIOSH) have termed Total Worker Health^®^ (TWH) [12]. TWH takes a holistic approach to advancing worker health that goes beyond wellness programs and instead takes institutional and governmental policies, programs, and practices into account [12]. One model for strategically and systematically increasing TWH programs and policies is through dissemination and implementation (D&I) science. D&I is based on the theory that research needs to have real-world implications for communities [12]. Furthermore, D&I science can help researchers better examine ‘what works’ for certain communities and the best environments for those interventions to be successful [12]. For example, trainings applied to commercial fishing settings have been shown to reduce injuries such as slips, trips, and falls overboard, but these programs can be difficult to implement because of researcher schedules, worker availability, seasonal demands, and the dynamic process of fostering trustworthiness and relationship building with commercial fishermen [13]. One solution for training implementation among a migratory population is through short message service (SMS) text messaging campaigns. A 2013 meta-analysis demonstrated that text messaging can be useful in other health promotion campaigns, as evident through 19 randomized clinical controlled trials in 13 countries [14], but SMS health promotion campaigns have yet to be implemented among commercial fishermen. Following D&I science best practices, our team studied not only *if* SMS texting could be implemented among commercial fishermen but also *how* contextual factors such as migratory populations and seasonal work impacted its feasibility.

In previous studies, our team showed that commercial fishermen are an under-reached population, rather than a hard-to-reach population, and that health promotion practices are requested by community members, such as dockside mobile health clinics (i.e., Docside Clinic), which can serve as an equitable intervention to improve health disparities, prevent injuries, and connect commercial fishermen to social services [14]. Research-to-practice/research-to-policy models have grown in popularity, but more empirical research on how D&I science is translated, disseminated, and implemented within occupational health is needed. Therefore, in this paper, we expand on previous research with commercial fishermen by demonstrating the methodology and implications of designing text message campaigns that aim to promote health and reduce injuries among high-risk occupations [15]. Moreover, we demonstrate the feasibility of SMS text messages as a tool for public health promotion in the commercial fishing industry.

### 1.1. Text Messaging in Health Promotion

Text messaging, or SMS campaigns, have grown in popularity as a communication tool for health promotion practices. Since its inception 40 years ago, mobile cellular telephones now have 8 billion users worldwide [16]. Within 20 years, researchers launched the first health promotion text campaign, which effectively helped teenagers manage their asthma [17]. This technology provides a tool for researchers to create health promotion campaigns that are scalable, accessible, easy to create, cost-effective, and can be tailored and targeted to specific populations [14]. Program initiatives have had diverse impacts, including fostering relapse prevention programs for people with schizophrenia and schizoaffective disorder, increasing engagement with cardiovascular disease prevention programs for rural participants, connecting rural and community patients to health care services during the COVID-19 pandemic, preparing expecting mothers for motherhood, increasing access to sexual services for at-risk youths, and enhancing cervical cancer screening awareness among African American women through spiritually sensitive messaging [18,19,20,21,22,23]. Not only has the technology been utilized as an extension of health care communication but also to implement prevention programs. For example, our team has used school-based text messaging campaigns to promote healthy relationships among teenagers [24]. Moreover, the use of text messages for health promotion is not limited to campaigns. Public officials also utilize SMS-based messaging to issue community emergency alerts [25]. While text messaging is an effective method of rapidly communicating lifesaving information, there is a gap in research on the use of these campaigns to prepare people for future emergencies. Furthermore, there are limited studies investigating the design and feasibility of implementing text message campaigns in the field of occupational health and safety.

### 1.2. Occupational Health and Safety Promotion and Commercial Fishermen

The precarious work of commercial fishing has been the target of workplace safety interventions and policies to promote interventions such as mandatory safety trainings in Nordic countries [26,27]. Non-digital promotion strategies utilized with commercial fishermen include hands-on safety training and the engagement of collaborative practices for implementing safety programs [28]. Captains report trainings that are periodic, current, practical, taught in the primary language of the fishermen, and culturally appropriate are important factors to improving occupational health and promoting safety [29]. Tellingly, among a group of Vietnamese fishermen in the GoM, close to half had concerns about how to use safety equipment on the vessel [13].

NIOSH recommends using health communications to translate research into products or tools that can be directly used by fishermen to reduce injuries and fatalities [30]. Currently, text campaigns have not been widely adopted to improve conditions within the commercial fishing industry. Recognizing that text campaigns have become a utilized platform to reach different populations for health promotion, we sought to apply this technology to commercial fishing. To address this gap in the literature, we developed a novel occupational health text campaign to provide insights and recommendations on the design and feasibility of a text campaign among commercial fishermen.

## 2. Materials and Methods

### 2.1. Text Messaging Campaign Design

Our team designed and implemented an 18-week texting campaign in the spring of 2021. After the initial recruitment, messages were delivered over the course of 18 weeks, with a total of 54 messages (3 messages per week) sent to fishermen. The spring was chosen as the start time, because it allowed the team time to pilot the messages before the commencement of the Texas shrimping season in offshore waters (typically 15 July) and the Atlantic hurricane season (beginning 1 June). The campaign was developed using a messaging service (i.e., Mobile Commons) and involved sending messages to commercial fishermen with a focus on preventing injury from slips, trips, and falls, as well as hurricane preparedness. Message delivery happened three times per week at approximately 4 p.m. CST. Although the campaign was not designed to be responsive, a research coordinator monitored the messages twice per day in the event a participant freely texted an emergency response (e.g., I need help). A fluent Spanish- and Vietnamese-speaking research coordinator, as well as a licensed psychologist and physician, were on stand-by in the event the person monitoring the campaign needed assistance. These instructions were verbally explained to the staff, as well as outlined in an emergency protocol that was emailed and kept in a cloud drive that all staff could easily access.

The campaign was designed to provide knowledge (e.g., ‘what to do when a hurricane is coming’), prevention (e.g., ‘how to plan for and navigate emergency conditions’), and resources (e.g., ‘where to receive more assistance regarding local occupational health promotion and emergency preparedness resources’) about occupational and hurricane safety. The campaign messages were designed using evidence-informed materials (i.e., US Coast Guard Trainings) that were identified and synthesized by our team. The concise messages were crafted with an emphasis on accessible and equitable communication practices and drew from real-world examples of fatal and non-fatal injuries of commercial fishermen. The study was focused on the design and feasibility of the SMS messaging campaign, and therefore, effectiveness results were not collected. Institutional review board (IRB) approval was obtained for this study.

### 2.2. Text Messaging Campaign Implementation

Fishermen could enroll in the trilingual campaign by texting ‘FISH’, ‘PESCADO’, or ‘C.A.’ to the short phone number for English, Spanish, or Vietnamese, respectively. The message content was multimodal. The messages contained written messaging and a link that would redirect the participant to an audio-based message. To ensure the equitable distribution of the campaign to all reading levels, written messages were designed for 6th–8th grade reading levels, and audio-based messages were included to ensure that illiterate fishermen could access them. The messages were encoded to Global System for Mobile Communications (GSM-7) character standards, which allows SMS messages to carry 160 characters per message. The messages were translated into Spanish and Vietnamese by certified translators. Native English, Spanish, and Vietnamese speakers recorded the audio message. The narrator emphasized communicating with clarity, confidence, and mindful tone and pace. The messages were recorded with professional audio standards and processed to maintain the utmost quality upon delivery. Hyperlinks for the audio messages were shortened through link-shortening software. This enabled the audio link to be included with fewer character counts and to save the character counts for the written message.

### 2.3. Text Message Design

The text messages were knowledge-, prevention-, or resource-based (Table 1). Knowledge messages provide a fact, explanation, or conveyed information. For example, after fishermen enrolled in the campaign, they received the message ‘In preparation for a hurricane, assemble a Go-Kit with items you cannot do without during an emergency. For an example of a Go-Kit, reply MORE.’ A fisherman seeking more information would then receive a subsequent message stating ‘Your kit should include water, nonperishable food, flashlights, a radio, first aid kid, batteries, whistle duct tape and moist towelettes’. A prevention message provided information to protect oneself, community, or property. A self-protective message was ‘If you are going to move your boat prior to a hurricane, determine a safe place to go and how long it will take for you to get there ahead of time.’ A response would follow when prompted stating ‘Check with your dock manager for their policy for hurricanes. Violating the safety zone could result in a fine.’ A resource-based message provided advice and assisted people in locating resources. An initial resource-based message was ‘Learn your docks Harbor of Safe Refugee Policy. In the event of a hurricane have a plan for where you can more your vessel. Reply MORE for details.’ A detailed message to provide avenues for more direct assistance would follow, stating ‘Contact the Texas Sea Grant Program for a more specific program for what you can do in a hurricane’ with the contact information included. No response was required to continue through the entire scheduled text campaign. Additionally, the opt-out for participants was kept simple to abide by federal regulations, as well as to ensure participants did not feel coerced to participate. To stop receiving messages, participants could text ‘STOP’, ‘ALTO’, or ‘DỪNG LẠI’ at any time.

### 2.4. Participants

We piloted the intervention among a subset of fishermen to test design and feasibility. Participants consisted of commercial fishermen throughout Southeast Texas and the Rio Grande Valley and were predominantly Vietnamese (*n* = 16) and Spanish (*n* = 5) im/migrant male adults. Participation in the occupational health campaign was voluntary. Recruitment occurred over a two-month period totaling 11 h and was based on relationship and trust building at the docks. First, relationships with dock managers were formed, and permission was granted for the research team to access the private docks. Dock managers identified the commercial fishermen, and then, snowball sampling was used. Research members would wait at the docks and converse with the fishermen. Typically, this was done earlier in the morning, as the heat and fishermen’s work commitments increased throughout the day. Additionally, trust and rapport had been previously built from community-based participatory research and implementing a free Docside Clinic among a group of Southeast Texas commercial fishermen [15].

**Figure 1:**
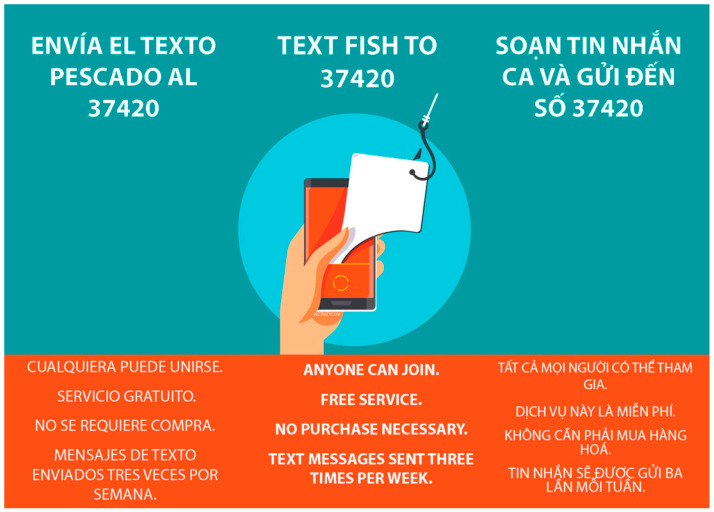
Trilingual text campaign flyer that was distributed to commercial fishermen and dock workers.

Trilingual visual aids were distributed at the docks and to participants, which provided information on how to enroll in the text campaign (see Figure 1). Waterproof flyers with large print were distributed to dock managers and dock workers, advertising the free texting campaign. Free t-shirts were distributed to anyone at the docks who wanted one, which included information on how to enroll in the text campaign. No incentives were provided for the fishermen who opted in. The text campaign did not ask for sensitive information that could pose risks to the participants, such as citizenship status.

## 3. Results

To our knowledge, our team was the first to disseminate and implement a health promotion text messaging campaign with GoM commercial fishermen. Our team found the challenges of disseminating and implementing the campaign to be more considerable than originally expected, especially since we had successfully implemented a text messaging campaign among high school students in a low socioeconomic status (SES) school district [24]. Considering the benefits of using a text messaging campaign to enhance health and prevent injuries among commercial fishermen, barriers to practice may inhibit its usefulness in certain situations. Therefore, we outline below the major areas of concern (i.e., implementation predictors) when designing a text messaging campaign for migratory populations that can benefit future researchers or practitioners (see Table 2). The lessons learned included the difficulty of navigating federal regulations regarding limits for special characters (i.e., Vietnamese diacritical marks), technology access, notable generational digital skills and technology use preferences, enrolling migratory participants, and navigating areas with limited cellular service. The implementation outcomes were not assessed at this stage, since we were conducting a feasibility study, but future effectiveness research should also consider adoption, fidelity, cost, penetration, and sustainability [31].

### 3.1. Trilingual Digital Communications and Navigating Federal Regulations

We encountered difficulty navigating structural factors such as federal regulations regarding limits for special characters. Considerable efforts were made to circumnavigate character count restraints that would not impact the message’s communication, understanding, and interpretation. It was imperative to ensure that the message content between the three languages communicated the same overall objective. Text contraction and expansion occurred when translating English messages into Spanish and Vietnamese. This resulted in differing amounts of words for a message in one of the three languages. Furthermore, following the GSM-7 character coding standard used for SMS messages, the message had to ultimately be 7 bits or 160 characters. In GSM-7 coding, not all characters, also known as code points, are coded equally. Developed in Europe for the English language, GSM-7 coding also supports most Latin-based characters, which facilitates easier use with Spanish messages. Yet, if a message includes any non-GSM character (i.e., accents), SMS automatically switches the messages to Universal Coded Character Set (UCS)-2 encoding, which limits the message body to 70 characters from 160 characters. For the Vietnamese, this had a powerful impact on digital health literacy. The initial messages within the text messaging campaign platform removed all accents from the Vietnamese messages. Notably, the 29 letters of the Vietnamese alphabet include 7 letters with four different diacritics (e.g., ă, â/ê/ô, ơ/ư, and đ) and 5 letters to convey tone (e.g., à, á, ả, ã, and ạ). Being a tonal language, different tonal inflections can convey different meanings. Subsequently, removing diacritics changes pronunciation, and altering tone changes the overall word meaning. Subsequently, messages had to be considerably recrafted to maintain as consistent communication standards as possible. In contrast, both English and Spanish are non-tonal languages supported by GSM-7, so developing the messaging platform was easier and quicker, though Spanish messages also had to be edited to ensure all accents were removed without changing the meaning of the word or phrase.

### 3.2. Technical Barriers

Enrollment of participants occurred while fishermen were docked and at port. Some participants were able to enroll through other means, such as word of mouth, noticing the flyers, or seeing the shirts that were passed out. When the research team was at the docks for participant enrollment, many technical barriers were made apparent.

Although cell phones are perceived as ubiquitous, access to and the use of a cell phone varied among commercial fishermen. Some fishermen could not participate, because they did not have a phone. Other fishermen had a pay-as-you-go plan and did not want to enroll in a messaging campaign that would utilize their monthly limits. Furthermore, some fishermen had phones but did not have data plans. Broadly, internet access was a challenge for those without a data plan, because there was no work Wi-Fi. When able to access Wi-Fi, they primarily used Facebook Messenger and WhatsApp to communicate. At the time of the campaign, our platform could not send SMS messages to these applications. Additionally, at one dock where recruitment occurred, a fisherman could not enroll because their device was not charged, and there were no outlets to securely charge his phone while working. One captain who originally enrolled in the campaign dropped his phone overboard. After securing a replacement phone with a lanyard, he lost it when getting off the vessel.

Since a major barrier was access to technological devices, teams need to consider factors that can influence who has access to the innovation being implemented and to what degree, such as the range of access to devices and participants’ digital knowledge [31]. Back-up plans for fishermen unable to participate using phones could help connect fishermen with digital resources. For example, teams could partner with programs that work to close digital divides by providing cell phones, Wi-Fi hotspots, and digital literacy workshops. These steps can ensure participants are not digitally excluded and aid participation. Prioritizing D&I approaches can help improve occupational health campaigns by focusing on adaptive and equitable strategies to ensure hard-to-reach and hardly reached workers are able to receive information and resources to improve their occupational health [31].

### 3.3. Generational Digital Skills

Generational differences were apparent in the digital divide as well. Many older fishermen did not use the texting options on their phones and primarily used voice call. Others that wanted to enroll in the campaign needed help texting the number to start the campaign. This ranged from finding their messaging application within their phone to texting a number not saved in their phone. Having Vietnamese and Spanish-speaking team members was critical to ensure participants could enroll without problems.

With a growing push towards digital-first health promotion platforms, providing high-quality and equitable service alternatives should be considered. Alternatives for participants preferring alternative texting platforms may include utilizing automated voice calls with interactive voice options or designing printable pamphlets with similar messaging and content as the text campaign.

### 3.4. Migratory Considerations

Tailoring an occupational health text campaign towards migrant workers should be adapted to reflect contextual factors such as a seasonal labor force and transient work, such as offshore, onshore, and different docks. Yet, once enrolled, the ability to communicate through scheduled text messages is an unparalleled outreach practice compared to those limited by timing, place, and in-person contact. While regulatory initiatives have been aimed at making commercial fishing more sustainable, it has increased pressure to prioritize operating in conditions that are less than ideal, such as having dynamic windows of opportunity during dangerous weather to earn income [32]. Understanding the regulatory impacts to the commercial fishing season, as well as seasonal migration patterns for certain workers, is important in the timing of one’s campaign and participant recruitment. Having an adaptable team that can meet workers where they are is important.

### 3.5. Timing of Messages

Enrollment at the dock varied. Depending on the time of day, week, or month, there was a range of the number of workers at the dock. Additional consideration should be given to the impact of message delivery when fishermen are offshore but enrolled in a campaign. Phone service is scattered and unpredictable offshore, with days to weeks on end without phone service. This results in a backlog of messages to be delivered once the fishermen have made it to shore. It is unclear how this might impact one’s interpretation and engagement with the messages. Pilot projects could be utilized to determine the extent of these implementer factors and how they may impact the adoption, use, and maintenance of text messaging campaigns to improve health disparities [33].

Compared to other text messaging campaigns, the ability to recruit and coordinate follow-up is dynamic and based around intermittent worker availability. To manage the dynamic nature of migration, we recruited and followed up with workers at a time when the docks had more workers present since they had to prepare their boats and equipment for the forthcoming season. Given that the population of commercial fishermen in Texas is primarily im/migrant workers, employment can vary depending on the fishing season and quotas, government policies towards H-2A visa holders, and market costs, such as the price of diesel compared to harvest profits.

## 4. Discussion

We conclude with short- and long-term suggestions for future technology interventions for under-reached worker populations, including ethical and policy regulations.

### 4.1. Short-Term Recommendations

Mobile technology is an important tool for mobile workers and offers workers an avenue to inform, empower, and connect in ways that previously could not occur. With technology becoming a main resource for one to interact with health care providers and to receive health care communication, researchers must recognize how its misuse can amplify structural racism and health care inequities. Policies focused on technology equity or ‘TechQuity’ must be prioritized as new technologies are adapted and utilized within health care practices [34,35]. Short-term policy programs could foster ‘tech-equity’ by increasing workers’ access to technological devices and literacy with such. Isolated workers, such as commercial fishermen, can utilize technology networks to resist exploitation, share and access resources, or receive health promotion materials. Utilizing technology-based communication tools must come with the recognition that these devices are subject to those who created them. We found this to be particularly relevant when crafting messages outside the GSM-7 recognized languages. As health care industries adapt to digital technologies, researchers should emphasize the public health need to end the digital divide. Further, technology should be crafted or updated to facilitate diversity, equity, and inclusion, with language, programming, and the use of artificial intelligence in development.

### 4.2. Long-Term Policy Recommendations

#### 4.2.1. Equitable Trainings

The US Coast Guard (USCG) is the principle federal agency that oversees the implementation of policies and regulations related to commercial fishing and aims to reduce vessel fatalities and accidents by conducting outreach, training, dockside vessel examinations, and at-sea vessel boardings [36]. Through our research and outreach with fishermen, we found that USCG representatives were genuinely concerned about the health and safety of fishermen and provided important resources that we adapted for the text campaign. However, USCG trainings are rarely offered in languages other than English. For example, their web-based trainings redirect a user to the CDC website. This information is presented only in English from the USCG, and the CDC is primarily in English, with not all resources translated into Spanish and no other language options presented to view the training [37]. Official government resources should consider the language and literacy needs of commercial fishing populations. Implementing SMS texting campaigns is one way that the USCG can offer resources even if personnel do not speak the language of the fishermen (e.g., Spanish or Vietnamese).

#### 4.2.2. Equitable Health Care Options

There is a critical need for reinvestment in the health of commercial fishermen and their families, especially because of the enormous risks that come with their occupation. The practice of government-provided health care to commercial fishermen and im/migrants is not new and extends 200 years with the Act for the Relief of Sick and Disabled Seamen. In fact, the health care of these groups is largely tied to the advancement of public health policy and practices. This Act led to the creation of federally controlled marine hospitals and the eventual development of the US Public Health Service Commissioned Corps. Under the Reagan Administration, continuous deregulation occurred towards government-sponsored medical coverage, and subsequently, there was decreased funding for the marine hospitals/U.S. public health hospitals that provided health care to commercial fishermen and im/migrants [38,39,40]. Moreover, restrictive policies towards Medicaid coverage currently limit im/migrant commercial fishermen from accessing health care coverage. Additionally, Affordable Care Act (ACA) marketplaces do not allow undocumented im/migrants to enroll in Medicaid or the Children’s Health Insurance Program (CHIP). While health promotion practices can increase health-seeking behaviors, evidence points towards social expenditures resulting in healthier populations [41]. Equitable health care options should be prioritized to enable historically marginalized workers and their families to access care.

## 5. Conclusions

A text message campaign was created to reduce work-related injuries among GoM commercial fishermen, with messaging focused on preventing injuries from slips, trips, and falls and fostering hurricane preparedness. With limited data on the design and feasibility of such campaigns, our team piloted a novel campaign using evidence-informed materials to enhance injury prevention. Our team designed and implemented a pilot trilingual text campaign with text and voice messaging. Piloting the intervention among a subset of fishermen allowed us to test the campaign’s accessibility and ease of use with the initial enrollment and subsequent utilization. This study applied D&I science to contextualize the factors that can impact the feasibility of a text campaign aimed at improving health disparities and emergency preparedness among commercial fishermen. Occupational health and safety promotion practices may benefit by adapting text message campaigns to target populations such as precarious workers, especially for populations that are under-reached or have dynamic schedules and work locations.

Authors note: Based upon previous research, we use the term fishermen because it is the preferred term among all genders and is considered gender neutral among the population

## Figures and Tables

**Table 1:** Text-Message Type.

	Knowledge	Prevention	Resource-Based
**Message Content**	Fact, explanation, or contained information	Information to protect oneself, community, or property	Advice and assisted people in locating resources
**Example of an Initial message**	‘In preparation for a hurricane, assemble a Go-Kit with items you cannot do without during an emergency. For an example of a Go-kit, reply MORE.’	‘If you are going to move your boat prior to a hurricane, determine a safe place to go and how long it will take for you to get there ahead of time.’	‘Learn your docks Harbor of Safe Refugee Policy. In the event of a hurricane have a plan for where you can more your vessel. Reply MORE for details.’
**Example of a response Message**	‘Your kit should include water, nonperishable food, flashlights, a radio, first aid kid, batteries, whistle duct tape and moist towelettes’	‘Check with your dock manager for their policy for hurricanes. Violating the safety zone could result in a fine.’	‘Contact the Texas Sea Grant Program for a more specific program for what you can do in a hurricane’ with contact information included.

**Table 2:** Data derived from Chaudoir et al. [12] and Dugan and Punnett [12,31].

Implementation Predictors	
Structural factors	Federal Regulations
Innovation factors	Technical Barriers
End user factors	Generational Digital Skills, Cell Phone Ownership
Organizational factors	Migratory Considerations
Implementer factors	Timing of Messages

## Data Availability

Data will be made available by the corresponding author upon responsible request.
